# Sleep and endocrine effects of acupuncture for insomnia: a systematic review and meta-analysis of randomized controlled trials

**DOI:** 10.3389/fmed.2026.1807826

**Published:** 2026-04-07

**Authors:** Yun Hu, Yi Liu, Wenwen Li, Yuting Wang

**Affiliations:** 1 Department of Traditional Chinese Medicine, Sir Run Run Hospital, Nanjing Medical University, Nanjing, China; 2 Department of English, College of International Studies, Guangzhou, China; 3 Department of Nephrology, Sir Run Run Hospital of Nanjing Medical University, Nanjing, China; 4 Department of Traditional Chinese Medicine, Suzhou BOE Hospital, Suzhou, China

**Keywords:** acupuncture, endocrine, insomnia, meta-analysis, systematic review

## Abstract

**Background:**

Insomnia, a prevalent sleep disorder linked to endocrine dysfunction, severely impairs quality of life. Acupuncture has been widely applied for its management, but evidence on its impact on sleep outcomes and endocrine regulation remains uncertain.

**Methods:**

Randomized controlled trials (RCTs) published until August 13, 2025, were explored in the subsequent databases: PubMed, Embase, Web of Science, Cochrane Library, China National Knowledge Infrastructure (CNKI), and Wanfang. Two reviewers independently screened studies, extracted data, and assessed risk of bias with RoB 2. Eligible trials enrolled insomnia patients receiving acupuncture or combined therapy versus sham acupuncture or medication. Outcomes included sleep-related scales, polysomnographic measures, and endocrine indicators. Meta-analysis was conducted using RevMan 5.4.

**Results:**

Twelve RCTs were included. Serum melatonin was higher with acupuncture based interventions than with controls (MD = 5.29, 95% CI: 3.01 to 7.57, *I^2^* = 99%), and the electroacupuncture subgroup showed a consistent increase (MD = 0.66, 95% CI: 0.34 to 0.98, *I^2^* = 0%). Salivary melatonin did not differ between groups, whereas salivary cortisol was lower in the acupuncture group (MD = 0.05, 95% CI: 0.02 to 0.08). Acupuncture reduced PSQI scores (MD = 3.39, 95% CI: 4.20 to 2.59). ISI improved in sham controlled trials (MD = 1.82, 95% CI: 2.95 to 0.68) but not in medication controlled comparisons. Polysomnography indicated improved sleep efficiency (MD = 6.40, 95% CI: 4.93 to 7.86) and overall improvements in total sleep time, sleep onset latency, and wake after sleep onset, with subgroup patterns suggesting contributions from treatment intensity and clinical subtype.

**Conclusion:**

This study suggests that acupuncture significantly improves subjective sleep quality, while also modulating melatonin and cortisol levels, highlighting its potential role in sleep and endocrine regulation.

**Systematic review registration:**

PROSPERO registration number is provided as CRD420251125885.

## 1 Introduction

Insomnia is among the most common sleep disorders encountered in clinical practice and is characterized by difficulty initiating sleep, frequent nocturnal awakenings or early morning awakenings, poor sleep quality, and impaired daytime functioning (1). Epidemiological studies suggest that approximately 10–20% of adults worldwide experience chronic insomnia symptoms, with prevalence estimates in some Chinese populations approaching 19% (2). Chronic insomnia carries wide ranging consequences. Beyond daytime fatigue, cognitive impairment, and increased vulnerability to mood disorders, it has also been associated with endocrine and metabolic disturbances, including altered cortisol secretion, dysregulated glucose and lipid metabolism, and perturbations in sex hormone profiles (3–5). Mechanistically, these alterations have been linked to activation of the hypothalamic–pituitary–adrenal (HPA) axis, heightened sympathetic tone, and proinflammatory signaling pathways (6, 7). Accordingly, insomnia should not be viewed solely as a sleep complaint but may reflect broader systemic dysregulation. In recent years, growing attention has focused on the sleep–neuro–endocrine–immune network, which posits that heightened neural excitability, stress hormone fluctuations, and inflammatory activation are interconnected processes that jointly disrupt physiological homeostasis in insomnia (6–8). Within this framework, interventions that improve sleep while attenuating endocrine dysregulation may be clinically relevant and mechanistically informative.

Acupuncture is commonly employed to address numerous clinical issues, especially those associated with pathological alterations in neuroendocrine function, including menopause, depression, and insomnia (9). By stimulating specific acupoints on the body, acupuncture may help modulate central and peripheral regulatory processes involving both the brain and the cardiovascular system (10). Several randomized controlled trials (RCTs) and systematic reviews have attempted to evaluate the efficacy of acupuncture in treating insomnia. An early systematic review indicated that acupuncture offers some improvement for insomnia, but also highlighted issues such as small sample sizes, difficulties in fully implementing blinding, and significant heterogeneity among existing studies (11). More recent evidence suggests that acupuncture may improve sleep quality and has an acceptable safety profile (12, 13). For instance, an RCT in perimenopausal women with insomnia reported that acupuncture significantly improved sleep efficiency and total sleep time (14). However, most of these studies primarily focused on subjective sleep outcomes, while relatively few have investigated objective biological markers such as endocrine indices, cortisol levels, or sex hormones. From a mechanistic perspective, both animal and human studies have attempted to elucidate the neuroendocrine regulatory effects of acupuncture. For example, some investigations have suggested that acupuncture may improve sleep architecture by modulating pathways involving cortisol and HPA axis activity (7, 15, 16). Additionally, conceptual and mechanistic reports have proposed that acupuncture may contribute to endocrine homeostasis by regulating key components of the neuroendocrine immune network, such as pro and anti inflammatory cytokines and stress related hormones (17, 18). However, studies that simultaneously incorporate acupuncture, insomnia, and endocrine function improvement within RCT designs and synthesize such evidence through meta analytic approaches remain limited.

Here, we aimed to systematically synthesize and quantitatively analyze evidence from RCTs regarding the use of acupuncture to ameliorate endocrine disturbances associated with insomnia. Through this work, we sought to establish a more reliable evidence base for the potential role of acupuncture in the management of insomnia with concomitant endocrine dysregulation. Such evidence may not only facilitate clinical decision making regarding its feasibility as an adjunctive or alternative therapy but also provide guidance for future mechanistic investigations, thereby advancing interdisciplinary research on acupuncture in sleep and endocrine metabolic regulation.

## 2 Methods

### 2.1 Study registration

This meta-analysis has been documented with the International Prospective Register of Systematic Reviews (PROSPERO; registration number: CRD420251125885) and fully complied with the Preferred Reporting Items for Systematic Reviews and Meta-analyses (PRISMA) (19).

### 2.2 Search strategy

The search was conducted across several electronic databases, including PubMed, Embase, Web of Science, Cochrane Library, China National Knowledge Infrastructure (CNKI), and Wanfang. The publication timeframe examined spanned from the commencement of each database up until 13 August 2025. The search of the literature was conducted in both English and Chinese languages. The search terms utilized for all databases can be found in Supplementary Table 1.

### 2.3 Inclusion criteria

#### 2.3.1 Types of research

Randomized controlled trials (RCTs) published in English or Chinese were included. Eligible trials were required to report clear statistical methods and provide extractable data. Duplicate publications, animal studies, reviews, and case reports were excluded.

#### 2.3.2 Types of participants

Adults with insomnia as the primary complaint were eligible, including defined subpopulations in which insomnia was a key clinical feature. Insomnia diagnoses were based on established diagnostic criteria or clinician confirmed diagnosis. Studies enrolling non insomnia populations were excluded.

#### 2.3.3 Varieties of interventions

Any acupuncture intervention involving skin penetration was eligible, including manual acupuncture, electroacupuncture (EA), warm needling, and auricular acupuncture, including auricular intradermal needles. Control interventions included sham acupuncture, usual care or no treatment, placebo, pharmacotherapy, or other non-acupuncture therapies. To evaluate acupuncture as an adjunctive therapy, add on trials comparing acupuncture plus a co intervention with the same co intervention alone were also included.

#### 2.3.4 Outcome measures

Endocrine markers were treated as the primary outcomes, with melatonin and cortisol as the prespecified key indicators. Measurements from serum or urine were eligible. Both post treatment values and change from baseline to endpoint were extracted, with post treatment values prioritized when both were available. Secondary outcomes included sleep related questionnaires and objective sleep parameters. Patient reported outcomes comprised the Pittsburgh Sleep Quality Index (PSQI) and the Insomnia Severity Index (ISI). Objective sleep outcomes included total sleep time (TST), sleep efficiency (SE), sleep onset latency (SOL), and wake after sleep onset (WASO), typically assessed by polysomnography or comparable objective tools.

### 2.4 Exclusion criteria

Studies were excluded if they met any of the following criteria: (1) non randomized design or unclear randomization procedures; (2) participants were not patients with insomnia or the reported outcomes were not insomnia related; (3) no endocrine outcomes were reported; (4) the effect of acupuncture could not be isolated because of unequal or non-comparable co interventions between groups; (5) inappropriate or inconsistent control conditions; (6) insufficient or non-extractable data, and the required information could not be obtained from the authors; or (7) duplicate publications, animal studies, reviews, or case reports.

### 2.5 Data extraction

Two reviewers independently conducted the literature search and study selection. After removing duplicates using EndNote, titles and abstracts were screened to identify potentially eligible records. The full texts of studies meeting the inclusion and exclusion criteria were then reviewed in detail to determine final eligibility. Disagreements were resolved through discussion, and, when necessary, consultation with a third reviewer.

### 2.6 Assessment of risk of bias

Methodological quality was assessed using the Cochrane risk of bias tool for randomized trials, RoB 2.0 (20). The assessment covered bias arising from the randomization process, deviations from intended interventions, missing outcome data, measurement of outcomes, and selection of the reported results. Each domain was rated as low risk of bias, some concerns, or high risk of bias.

### 2.7 Statistical analysis

The analysis was performed utilizing RevMan 5.4 software. Continuous results were expressed as mean differences (MD) along with a 95% confidence interval (CI). A *p*-value of less than 0.05 was deemed statistically significant. The *I^2^* test was employed to evaluate heterogeneity. A random effects model was used when *I^2^* exceeded 50%, while a fixed effects model was adopted in other cases. Subgroup analyses were conducted according to various control methods. If at least 10 trials were available, we intended to assess publication bias using a funnel plot.

## 3 Results

### 3.1 Literature search

**Figure 1:**
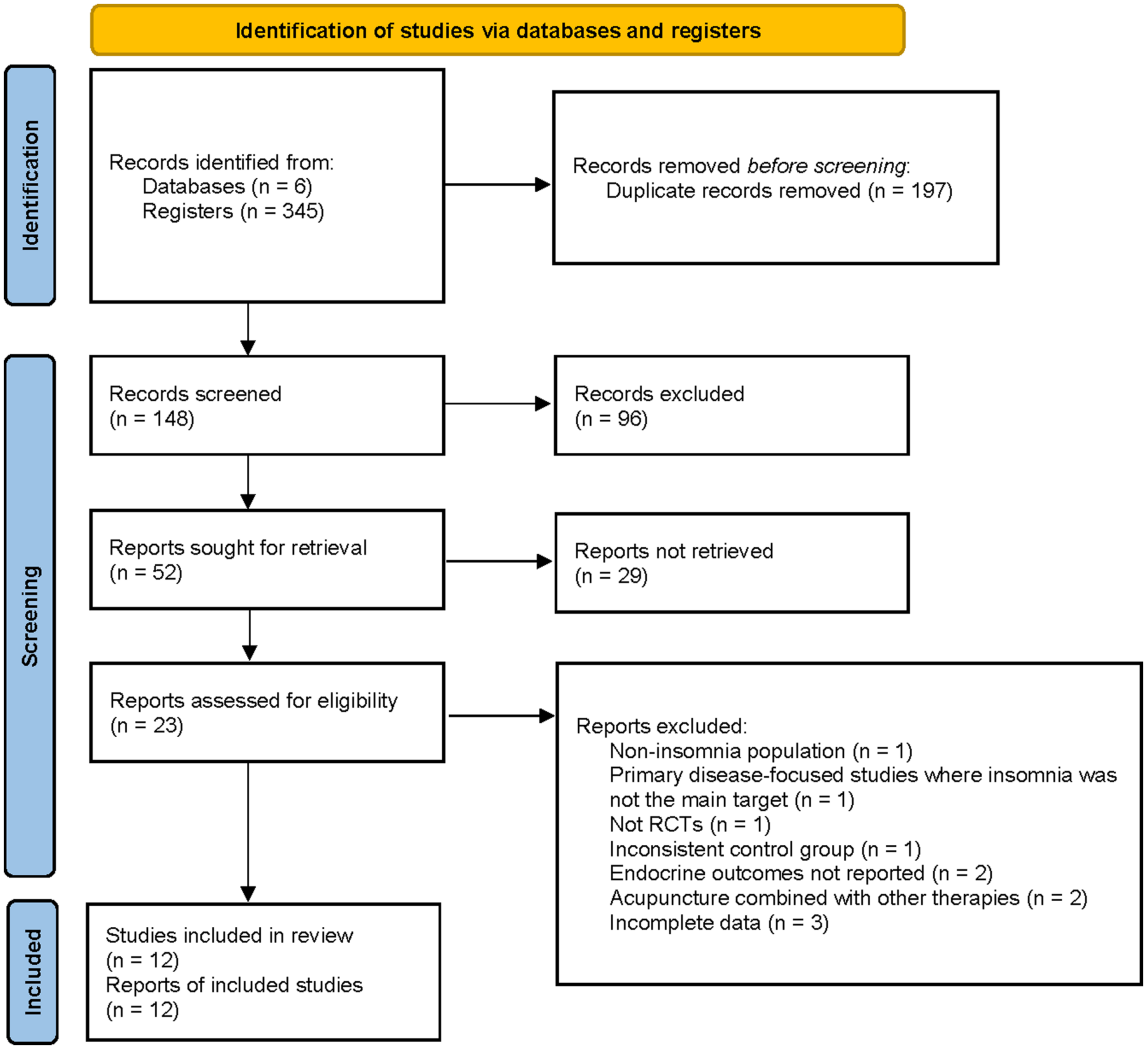
Study of flow diagram.

As shown in Figure 1, a total of 345 records were identified from electronic databases and trial registers. After removal of 197 duplicates, 148 records remained for screening. Of these, 96 were excluded after title and abstract screening. Full texts were sought for 52 studies, but 29 could not be retrieved. The remaining 23 reports were assessed for eligibility, and 11 were excluded for the following reasons: non insomnia populations (*n* = 1), insomnia not being the primary focus (*n* = 1), non RCT design (*n* = 1), inappropriate or inconsistent control group (*n* = 1), absence of endocrine outcomes (*n* = 2), inseparable combination of acupuncture with other therapies (*n* = 2), and incomplete data (*n* = 3). Ultimately, 12 randomized controlled trials met the inclusion criteria and were included in the systematic review and meta analysis.

### 3.2 Characteristics of the studies

As shown in Tables 1, 2, the included trials covered a broad range of insomnia related conditions, including elderly insomnia, chronic insomnia, pregnancy related insomnia, cancer related insomnia, insomnia associated with cardiovascular disease, and delayed sleep wake phase disorder. Sample sizes ranged from 14 to 150 participants, and the mean age was generally between 40 and 70 years. Interventions included electroacupuncture, manual acupuncture, warm needle moxibustion, and auricular intradermal press needles. Across protocols, commonly used acupoints clustered around GV20 with EX HN3 or GV29, together with HT7, SP6, and PC6, and several studies also incorporated back shu points such as BL15 and BL23. Electroacupuncture protocols typically applied low frequency stimulation at 2 Hz or 4 Hz with a session duration of 30 min. Overall treatment schedules ranged from 2 to 7 sessions per week and lasted 3 to 8 weeks, with most trials using a 4 week course and 20 to 30 min per session. Control conditions primarily included sham needling procedures, conventional pharmacotherapy, or usual care, and several trials used an add on design comparing acupuncture plus medication with medication alone. Outcomes included subjective measures such as PSQI, ISI, and AIS, as well as polysomnography derived parameters including TST, SE, SOL, and WASO. In addition, several endocrine outcomes were assessed, including melatonin, cortisol, ACTH, norepinephrine, and leptin. Collectively, these variations provided a comprehensive dataset for meta analysis, while also indicating potential sources of heterogeneity across studies.

**Table 1:** Characteristics of the included studies.

Study	Patient condition	Sample size (E/C)	Sex (male/female)	Mean age (E/C)	E			C			Outcomes
					Type	Frequency	Duration	Type	Frequency	Duration	
Wang (2021) (21)	Insomnia in the elderly	30/29	24/35	69 ± 4/69 ± 5	EA	3 sessions/week	4 weeks	Sham EA	3 sessions/week	4 weeks	PSQI; Serum MT
Wang et al. (2025) (23)	Chronic insomnia	50/50	40/60	52.26 ± 9.08/51.25 ± 8.47	MA + Estazolam Tablet	6 sessions/week	4 weeks	Estazolam Tablet	1 mg, once daily at bedtime	4 weeks	PSQI; ISI; SOL; WASO; TST; SE; Serum CORT; Serum ACTH
Wu et al. (2021) (41)	Chronic insomnia	34/32	26/40	47.00 ± 13.59/46.22 ± 11.91	EA	3 sessions/week	4 weeks	Sham EA	3 sessions/week	4 weeks	PSQI; Serum CORT
Foroughinia et al. (2020) (25)	Pregnancy-related insomnia	26/29	0/55	31.7 ± 5.7/30.2 ± 3.4	MA	10 sessions over 3 weeks	3 weeks	Sham MA	10 sessions over	3 weeks	PSQI; Urinary 6-Sulfatoxymelatonin
Lee et al. (2020) (22)	Insomnia disorder	49/52	NR	51.78 ± 5.09/52.00 ± 4.96	EA	2–3 sessions/week	4 weeks	Sham EA	2–3 sessions/week	4 weeks	ISI; PSQI; SOL; WASO; TST; SE; Salivary MT; Salivary CORT
Lee et al. (2022) (42)	Cancer-related insomnia	8/6	7/7	57.63 ± 10.19/62.33 ± 15.8	EA	2–3 times per week	4 weeks	Sham EA	2–3 times per week	4 weeks	ISI; PSQI; SOL; TST; SE; Salivary MT; Salivary CORT
Mou et al. (2023) (43)	Delayed sleep–wake phase disorder	30/30	24/36	39 ± 7/39 ± 6	MA	3 times/week for first 4 weeks, then 2 times/week for next 4 weeks	8 weeks	Sham MA	3 times/week for first 4 weeks, then 2 times/week for next 4 weeks	8 weeks	PSQI; Serum MT
Liu et al. (2023) (44)	Insomnia in the elderly	60/60	66/54	68.11 ± 5.11/68.45 ± 5.11	Dexzopiclone Tablets +Acupuncture (Regulating Yin and Yang method)	6 times per week	4 weeks	Dexzopiclone Tablets	2 mg, once per day at bedtime	4 weeks	PSQI; Serum MT; Serum NE
Wu et al. (2022) (24)	Insomnia in patients with coronary heart disease	30/30	29/31	Not explicitly stated (Range: 42-75/C: 45–79)	Auricular Intradermal Acupuncture+ Alprazolam	Alternating ears every other day	4 weeks	Alprazolam	0.4 mg, once per day at bedtime	4 weeks	PSQI; AIS; Serum MT; Serum Leptin
Wang (2025) (45)	Insomnia in patients during the remission stage of Bipolar Disorder	75/75	79/71	41.35 ± 5.86/40.94 ± 5.73	Manual Acupuncture (Shugan Tiaoshen method) combined with Western Medicine	Once per day	8 weeks	Western Medicine only (Magnesium Valproate Tablets + Quetiapine Fumarate Tablets)	According to drug dosage guidelines	8 weeks	PSQI; TST; SOL; SE; WASO; Serum MT; Serumβ-Endorphin
Yang (2024) (46)	Chronic insomnia	50/50	36/64	56.65 ± 4.51/56.80 ± 4.45	Warming acupuncture+Oral Zolpidem	3 times per week, every other day	4 weeks	Oral Zolpidem	Once per night	4 weeks	PSQI; ISI; SOL; WASO; TST; SE; Serum MT
Xu et al. (2022) (47)	Elderly patients with insomnia	29/28	18/39	67 ± 3/67 ± 3	EA	3 times per week	4 weeks (12 sessions total)	Sham EA	3 times per week	4 weeks (12 sessions total)	PSQI; Serum MT

E, experimental group; C, control group; EA, electroacupuncture; MA, manual acupuncture; PSQI, Pittsburgh Sleep Quality Index; ISI, Insomnia Severity Index; MT, melatonin; SOL, sleep onset latency; WASO, wake after sleep onset; TST, total sleep time; SE, sleep efficiency; CORT, cortisol; ACTH, adrenocorticotropic hormone; NE, norepinephrine; AIS, Athens Insomnia Scale; NR, not reported.

**Table 2:** Details of interventions in included trials.

Study	Methods of intervention	Acupoints	Acupuncture skills	Dosage (key parameters)
Wang (2021) (21)	E: EA C: Superficial needling (2–3 mm) with no current	GV20, GV29, HT7, SP6, BL15, BL23	Depth/angle (EA): 10–20 mm; GV20 oblique insertion (~15°) into subgaleal loose connective tissue; GV29 transverse toward the nasal tip; HT7 and SP6 perpendicular; BL15/BL23 oblique toward the spine (~45°) EA parameters: Intermittent wave, 2 Hz; intensity set to patient tolerance; EA applied to GV20 and GV29.	Sessions per week: 3 sessions/week (every other day) Minutes per session: 30 min Treatment duration: 4 weeks
Wang et al. (2025) (23)	E: MA + estazolam C: Estazolam	EX-HN1, HT7, SP6, BL15, BL14, BL20, BL15, BL23, KI3, BL15, BL19, BL18, LR3, BL21, ST36	Manipulation: twisting method; main points used an even reinforcing–reducing technique Syndrome-specific manipulation on add-on points: reinforcing (heart–spleen deficiency; heart deficiency with timidity), reducing (liver yang rising), even method (spleen–stomach disharmony), and for heart–kidney disharmony: BL23 reinforcing + BL15 reducing.	Needle retention: 30 min; one additional needle manipulation during retention Treatment schedule: 6 consecutive treatment days followed by 1 rest day Concomitant medication (both groups): estazolam 1 mg nightly Treatment duration: 4 weeks
Wu et al. (2021) (41)	E: EA + sleep-hygiene education C: Sham EA + sleep-hygiene education	GV20, EX-HN3, HT7, SP6, BL13, BL15, BL18, BL20, BL23	Oblique needling 10–15 mm toward the Governor Vessel; tonifying manipulation at BL13/BL15/BL20/BL23 and reducing manipulation at BL18; no retention for these points GV20/EX-HN3 inserted along the GV direction (5–10 mm); HT7/SP6 perpendicular (~15 mm); Deqi obtained; even reinforcing–reducing twirling EA parameters: intermittent wave, 2 Hz; intensity to tolerance (reported as 2–5); 30-min retention; sham group connected but with intensity off	30 min/session; every other day (3 sessions/week); total 12 sessions; delivered by two acupuncturists with >5 years’ experience; Treatment duration: 4 weeks, follow-up at 4 weeks after treatment end (questionnaires only)
Foroughinia et al. (2020) (25)	E: MA + sleep hygiene education C: Sham (non-penetrating) acupuncture + sleep hygiene education	KI-3, HT-7, ST-36, PC-6, DU-20, GB-20, EX-8	Needles inserted at ~90° to the skin; rotated anti-clockwise; Manual manipulation to elicit De Qi; needles retained ~30 min; Delivered by an expert practitioner (>15 years’ experience)	Sterilized stainless-steel needles: 0.3 × 40 mm and 0.3 × 25 mm; 10 sessions/3 weeks (4 in week 1; then 3/week); ~30 min/session; Treatment duration: 3 weeks
Lee et al. (2020) (22)	E: EA + sleep-hygiene brochure C: Sham EA using Park Sham Device at non-acupoints; same schedule + brochure	GV20, EX-HN3, HT7, PC6, BL63, KI4	Needles: disposable filiform 0.25 × 40 mm; insertion depth 5–20 mm; De qi elicited before stimulation; EA: 30 min/session; continuous wave 4 Hz at ~80% intensity; Sham: PSD at 10 non-acupoints; device connected but deactivated (sound/light mimicked)	2–3 sessions/week; 30 min/session; total 10 sessions Treatment duration: 4 weeks
Lee et al. (2022) (42)	E: EA + sleep-hygiene brochure C: non-penetrating placebo needles at non-acupoints (same number of sites), deactivated EA device (sound/light only), otherwise same schedule as EA	GV20, EX-HN3, HT7, PC6, BL63, KI4	Needle manipulation: de qi achieved by twisting; Insertion depth: 0.2–1.5 cun	Needles: disposable sterilized, 0.25 × 25 mm; Electrical parameters: 4 Hz, continuous wave; retention/stimulation time: 30 min/session; Frequency: 2–3 sessions/week; total sessions: 10 Treatment duration: 4 weeks
Mou et al. (2023) (43)	E: Sleep hygiene education + manual acupuncture at fixed body points; C: Sleep hygiene education + sham acupuncture using placebo blunt needles (non-penetrating) at the same points	BL62, KI6, LI4, LR3, ST36, SP6	Manual needling used twirling manipulation to elicit de qi (soreness/numbness/heaviness/distension); Sham procedure used a placebo blunt, non-penetrating needle with a foam pad (no skin penetration / no de qi)	Needles (active): 0.30 × 40 mm; depth 0.3–0.5 cun and 1.0–1.5 cun; Sham device: 0.40 × 13 mm placebo blunt needle (non-penetrating); Session time: 20 min; Treatment duration: 4 weeks
Liu et al. (2023) (44)	E: Dexzopiclone 2 mg PO at bedtime (q.d.) + MA C: Dexzopiclone 2 mg PO at bedtime (q.d.)	CV4, KI6, GV4, GV14, GV20, CV12, BL15, KI3, BL23, BL20, LR3, BL18, BL21, ST36, GB40, PC7, BL19	Supine: skin disinfected with 75% ethanol; Deqi required; CV12 + CV4, twisting reinforcing manipulation ×60 s; retain 30 min; GV20 transverse insertion; GV4/KI6/GV14 perpendicular insertion; twisting reducing manipulation ×60 s; retain 30 min	Needle: 20 mm × 40 mm; manipulation 60 s after Deqi; Retention 30 min (two-step protocol); frequency 1/day, 6/week Dexzopiclone 2 mg qhs (1 mg/tablet); Treatment duration: 4 weeks
Wu et al. (2022) (24)	E: Conventional CHD treatment + auricular intradermal acupuncture (press-needle) C: Conventional CHD treatment + alprazolam 0.4 mg PO qHS, once nightly	TF4, AT4, AT3, CO15	75% ethanol disinfection; probe to identify the most tender/reactive point; Sterile press-needle inserted vertically; secured with tape; ears alternated	Press-needle: 0.22 mm × 1.50 mm; treatment every other day; alternate ears; Treatment duration: 4 weeks
Wang (2025) (45)	E: Magnesium valproate + quetiapine fumarate + MA, once daily; C: Magnesium valproate + quetiapine fumarate	DU20, EX-HN3, DU26, CV15, LI4, LR3, CV4, CV6, HT7, PC6, BL18, BL15	Reducing method with “bird-pecking” at DU20/EX-HN3/DU26/CV15 (~0.2 cun); Twirling/lifting–thrusting (reducing) at LI4/LR3 and HT7/PC6/BL18/BL15 (~0.4 cun); Even method at CV4/CV6 (45° oblique, ~0.4 cun); retention 30 min	Magnesium valproate: start 15 mg/kg/day (BID), titrate to 25 mg/kg/day within 4 weeks; continue to week 8; Quetiapine fumarate: start 50 mg/day (BID), titrate to 100 mg/day within 4 weeks; continue to week 8; Treatment duration: 8 weeks
Wang (2021) (21)	E: Sinox 10 mg PO qhs + warm-needle moxibustion at peri-umbilical four-side points; C: Sinox 10 mg PO qhs	CV8, GV20, PC6, HT7, EX-HN22	Peri-umbilical points: 45° oblique insertion (~35 mm) toward CV4, with twirling to elicit deqi; retain 30 min; GV20: subcutaneous insertion toward posterior (~25 mm), no manipulation; PC6/HT7/Anmian: perpendicular insertion (~25 mm), even reinforcing–reducing; numbness/soreness desirable	Sinox: 10 mg nightly; Warm-needle moxibustion: moxa segment ~2 cm on needle handle; cardboard protection; 2 moxa cones per point/session; Needle retention: 30 min; Treatment duration: 4 weeks
Wang et al. (2025) (23)	E: Sleep health education + EA; C: Sleep health education + superficial needling at the same acupoints and schedule; EA leads connected at GV20–EX-HN3 but wires were cut (no current)	GV20, GV24, EX-HN3, HT7, SP6	Head points: oblique insertion; body points: perpendicular insertion; participants rested with an eye mask during treatment	Needles: 0.30 × 40 mm; depth 10–20 mm (EA) vs. ~ 2–3 mm (superficial control); 30 min/session; EA at 2 Hz (intermittent wave) on GV20–EX-HN3; Treatment duration: 4 weeks (12 sessions)

E, experimental group; C, control group; EA, electroacupuncture; MA, manual acupuncture; PSQI, Pittsburgh Sleep Quality Index; ISI, Insomnia Severity Index; AIS, Athens Insomnia Scale; TST, total sleep time; SOL, sleep onset latency; SE, sleep efficiency; WASO, wake after sleep onset; MT, melatonin; CORT, cortisol; ACTH, adrenocorticotropic hormone; NE, norepinephrine; CHD, coronary heart disease; DU20, Baihui; DU26, Shuigou; GV4, Mingmen; GV14, Dazhui; GV20, Baihui; GV24, Shenting; GV29, Yintang; EX-HN1, Sishencong; EX-HN3, Yintang; EX-HN22, Anmian; EX-8, Anmian; HT7, Shenmen; PC6, Neiguan; PC7, Daling; SP6, Sanyinjiao; KI3, Taixi; KI4, Dazhong; KI6, Zhaohai; ST36, Zusanli; LR3, Taichong; LI4, Hegu; BL13, Feishu; BL14, Jueyinshu; BL15, Xinshu; BL18, Ganshu; BL19, Danshu; BL20, Pishu; BL21, Weishu; BL23, Shenshu; BL62, Shenmai; BL63, Jinmen; GB40, Qiuxu; CV4, Guanyuan; CV6, Qihai; CV8, Shenque; CV12, Zhongwan; CV15, Jiuwei; TF4, Auricular Shenmen; AT4, Auricular Subcortex; AT3, Auricular Occiput; CO15, Auricular Heart.

### 3.3 Risk of bias assessment

**Figure 2:**
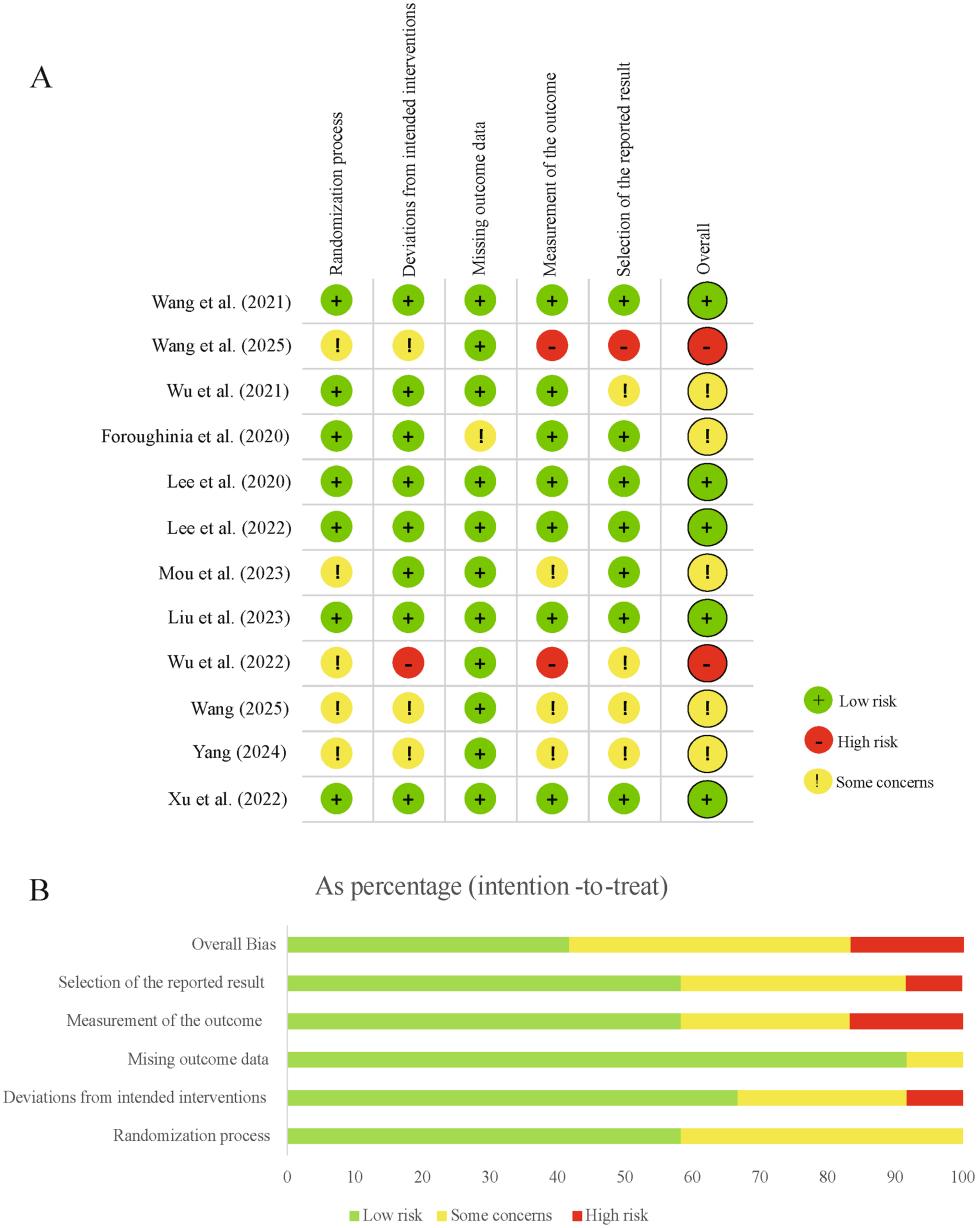
Assessment of bias risk for the studies included: **(A)** evaluation of literature quality and **(B)** summary of the quality assessment of the literature.

Figure 2 presents the risk of bias assessment for the 12 included RCTs. Most studies were judged to have a low risk of bias in randomization, intervention adherence, and outcome reporting. For example, Wang et al. (21) and Lee et al. (22) employed clearly described randomization procedures and appropriate control designs. However, several studies showed higher risks in outcome measurement. Wang et al. (23) relied primarily on self reported scales without sufficient objective assessments and was rated as high risk in this domain. Wu et al. (24) was judged as high risk because of incomplete outcome data. Some trials showed some concerns in selective reporting, including Foroughinia et al. (25), where secondary outcomes were not fully reported. These methodological features are consistent with the limitations summarized in Supplementary Table 2. Although most trials reported improvements in subjective sleep measures and, in several cases, endocrine markers such as melatonin and cortisol, common constraints included small sample sizes, single center designs, limited blinding procedures, short intervention periods, and lack of long term follow up. Taken together, the risk of bias evaluation and the identified methodological limitations suggest that although the overall direction of evidence favors acupuncture, heterogeneity in study design and variability in reporting quality warrant cautious interpretation of the pooled findings.

### 3.4 Assessment of endocrine-related outcome measures

**Figure 3:**
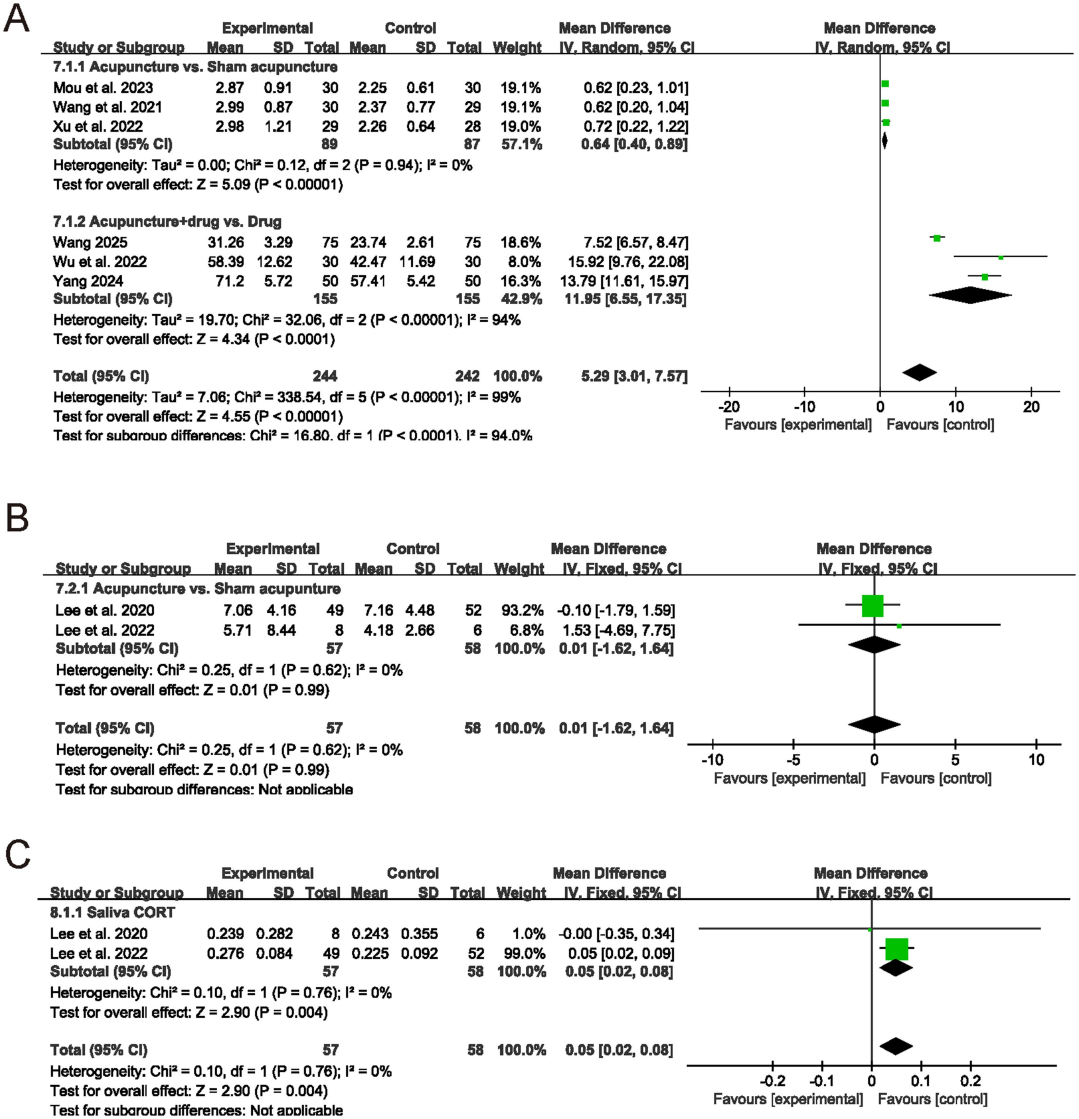
Forest plots for endocrine outcomes. Forest plots of **(A)** serum melatonin (MT), **(B)** salivary MT, and **(C)** salivary cortisol (CORT).

Six RCTs reported serum MT levels as the primary outcome. Pooled analysis showed higher serum MT with acupuncture-based interventions than with controls (MD = 5.29, 95% CI: 3.01–7.57), although heterogeneity was considerable (*I*^2^ = 99%) (Figure 3A). When stratified by comparator, trials of acupuncture versus sham acupuncture demonstrated a small but consistent increase in serum MT (MD = 0.64, 95% CI: 0.40–0.89; *I*^2^ = 0%), whereas trials of acupuncture plus medication versus medication alone showed a larger increase (MD = 11.95, 95% CI: 6.55–17.35; *I*^2^ = 94%). In a further technique-based subgroup analysis (EA vs. MA), EA yielded a homogeneous increase in serum MT (MD = 0.66, 95% CI: 0.34–0.98; *I*^2^ = 0%), while MA showed substantial inconsistency and was not statistically significant (MD = 4.06, 95% CI: −2.71–10.82; I^2^ = 99%) (Supplementary Figure 1). The EA subgroup showed a consistent, statistically significant increase in serum MT with no heterogeneity, suggesting a more reproducible endocrine effect than the MA subgroup in this evidence base. Additionally, as shown in Figure 3B, the meta-analysis of salivary MT included two studies. The pooled results indicated no significant difference between the acupuncture group and the sham acupuncture group in improving salivary MT levels (MD = 0.01, 95% CI: −1.62–1.64, *p* = 0.99). No heterogeneity was observed between the two groups (*I*^2^ = 0%, *p* = 0.62), indicating consistent results. As shown in Figure 3C, the meta-analysis of salivary CORT also included two studies. The pooled results indicated that the acupuncture group had significantly lower salivary CORT levels than the control group (MD = 0.05, 95% CI: 0.02–0.08, *p* = 0.004). Inter-study heterogeneity was extremely low (*I*^2^ = 0%, *p* = 0.76), indicating consistent and robust results. This suggests that acupuncture may have a positive effect on reducing salivary CORT and MT levels.

### 3.5 Assessment of PSQI score

**Figure 4:**
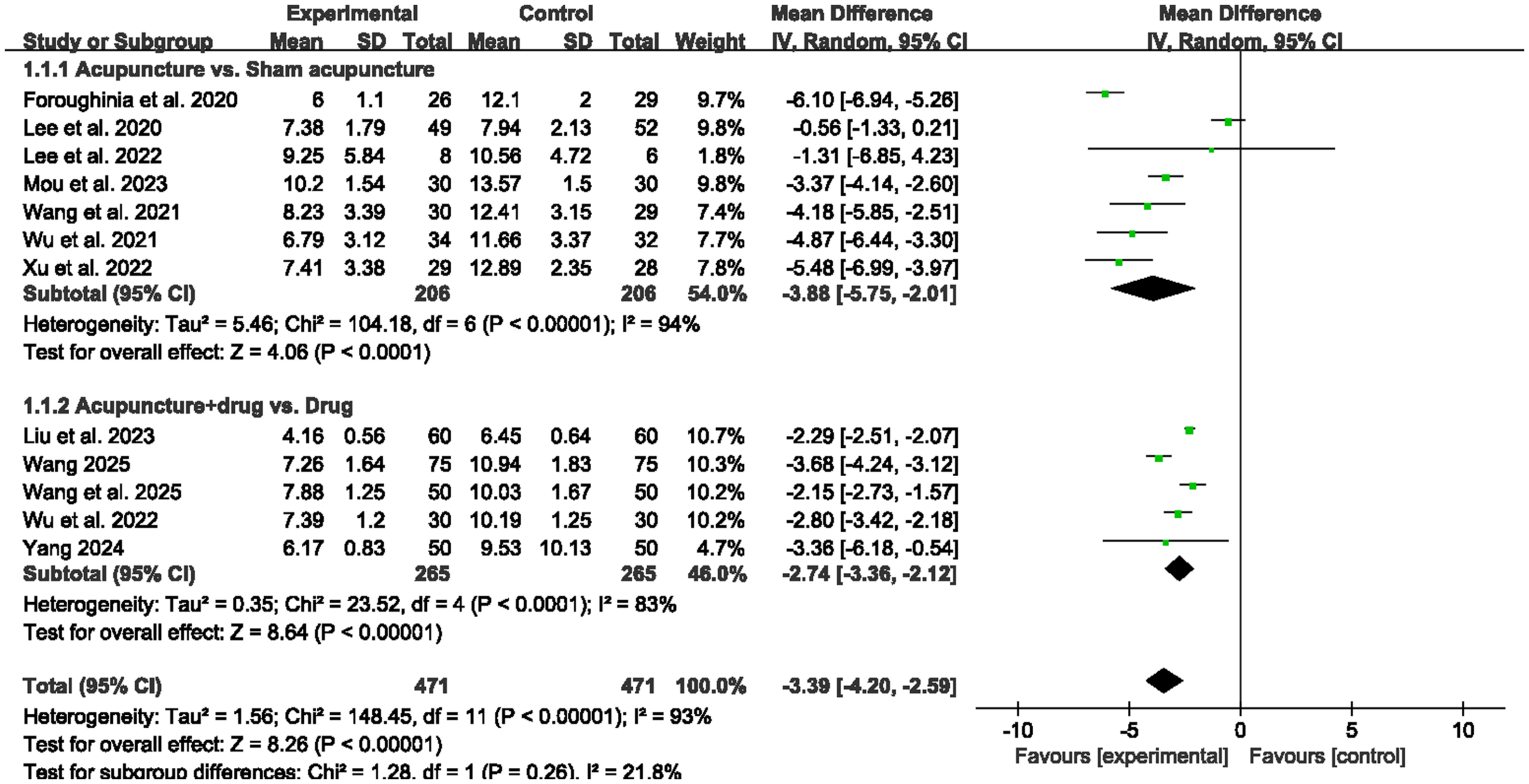
Forest plots for PSQI score.

A total of 12 randomized controlled trials were included to evaluate the effects of acupuncture on PSQI scores in patients with insomnia, with 471 participants in the acupuncture groups and 471 in the control groups (Figure 4). The pooled analysis showed that acupuncture significantly reduced PSQI scores compared with control conditions (MD = −3.39, 95% CI: −4.20 to −2.59, *p* < 0.00001). Subgroup analyses revealed that acupuncture compared with sham acupuncture produced a mean difference of −3.88 (95% CI: −5.75 to −2.01, *p* < 0.0001; *I^2^* = 94%), whereas acupuncture combined with medication compared with medication alone resulted in a mean difference of −2.74 (95% CI: −3.36 to −2.12, *p* < 0.00001; *I^2^* = 83%). Further subgroup analyses are presented in Supplementary Figure 2. By acupuncture technique, electroacupuncture and manual acupuncture showed comparable effects, EA (MD = −3.45, 95% CI = −5.99 to −0.92, *p* = 0.008, *I^2^* = 92%) and MA (MD = −3.48, 95% CI = −4.61 to −2.35, *p* < 0.00001, *I^2^* = 96%). By treatment duration, PSQI data were predominantly derived from 4 week interventions (MD = −3.37, 95% CI = −4.25 to −2.48, *p* < 0.00001, *I^2^* = 93%). By treatment frequency, the effect differed across subgroups, 3 sessions per week (MD = −5.16, 95% CI = −6.06 to −4.25, *p* < 0.00001, *I^2^* = 44%), 6 sessions per week (MD = −2.27, 95% CI = −2.47 to −2.07, *p* < 0.00001, *I^2^* = 0%), and 7 sessions per week (MD = −0.57, 95% CI = −1.33 to 0.18, *p* = 0.14, *I^2^* = 0%), with a significant subgroup difference (*p* < 0.00001). By insomnia subtype, both chronic insomnia (MD = −3.37, 95% CI = −5.37 to −1.37, *p* = 0.001, *I^2^* = 81%) and elderly insomnia (MD = −3.89, 95% CI = −6.05 to −1.72, *p* = 0.0004, *I^2^* = 91%) showed reductions in PSQI. Overall, acupuncture was associated with lower PSQI scores across comparator types and insomnia subgroups, while the magnitude of benefit varied by treatment frequency, suggesting that treatment intensity may contribute to differences in observed effects.

### 3.6 Assessment of ISI score

**Figure 5:**
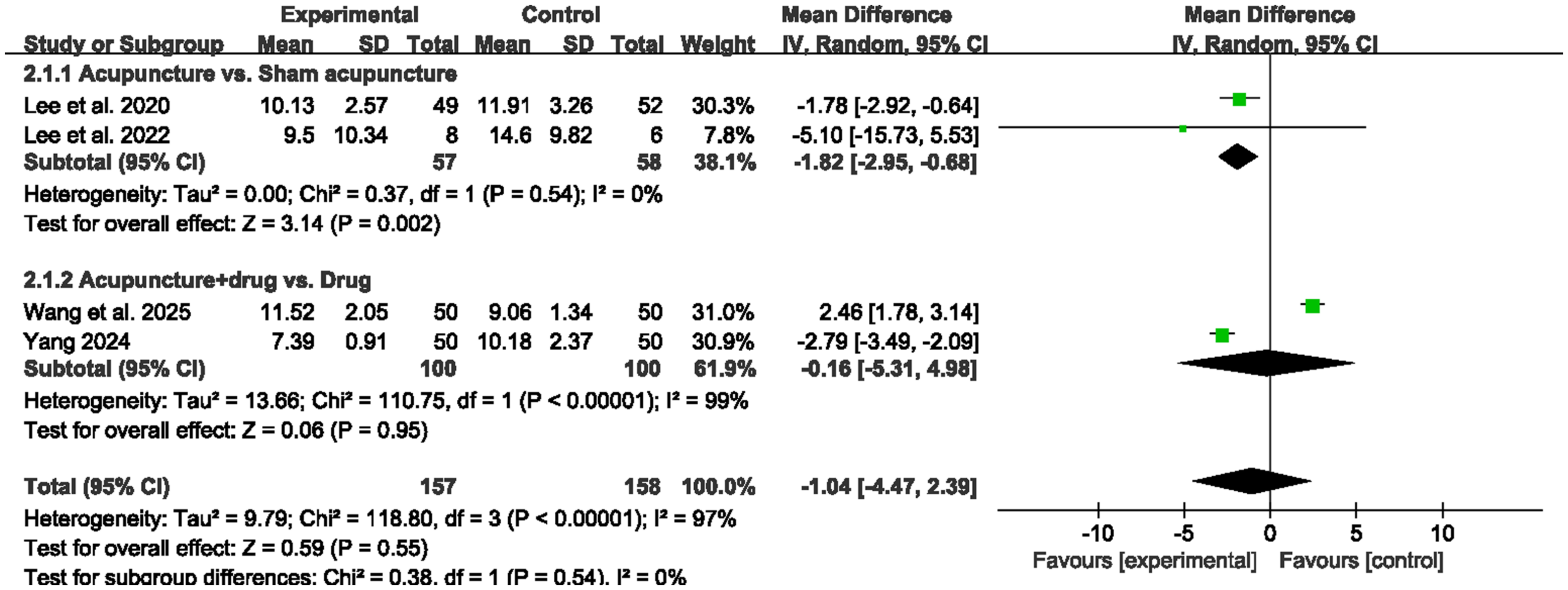
Forest plots for ISI score.

As shown in Figure 5, four randomized controlled trials were included for the analysis of ISI scores (157 participants in the acupuncture groups and 158 in the control groups). Subgroup analyses by comparator indicated that acupuncture versus sham acupuncture was associated with a significant improvement in ISI (MD = −1.82, 95% CI: −2.95 to −0.68, *p* = 0.002, *I^2^* = 0%), whereas acupuncture plus medication versus medication alone showed no significant difference (MD = −0.16, 95% CI: −5.31 to 4.98, *p* = 0.95, *I^2^* = 99%), with no evidence of a between subgroup difference (*p* = 0.54). In addition, further subgroup analyses are presented in Supplementary Figure 3. By acupuncture technique, the available data did not indicate a significant effect in the included studies (MD = −0.21, 95% CI: −4.14 to 3.72, *p* = 0.92, *I^2^* = 95%). By treatment duration, ISI data were derived from 4 week interventions (MD = −1.04, 95% CI: −4.47 to 2.39, *p* = 0.55, *I^2^* = 97%). By treatment frequency, acupuncture delivered twice weekly showed a significant reduction in ISI (MD = −1.82, 95% CI: −2.95 to −0.68, *p* = 0.002, *I^2^* = 0%). By insomnia subtype, the chronic insomnia subgroup did not show a significant difference (MD = −0.07, 95% CI: −0.56 to 0.42, *p* = 0.78, *I^2^* = 99%).

### 3.7 Assessment of polysomnographic sleep outcomes

**Figure 6:**
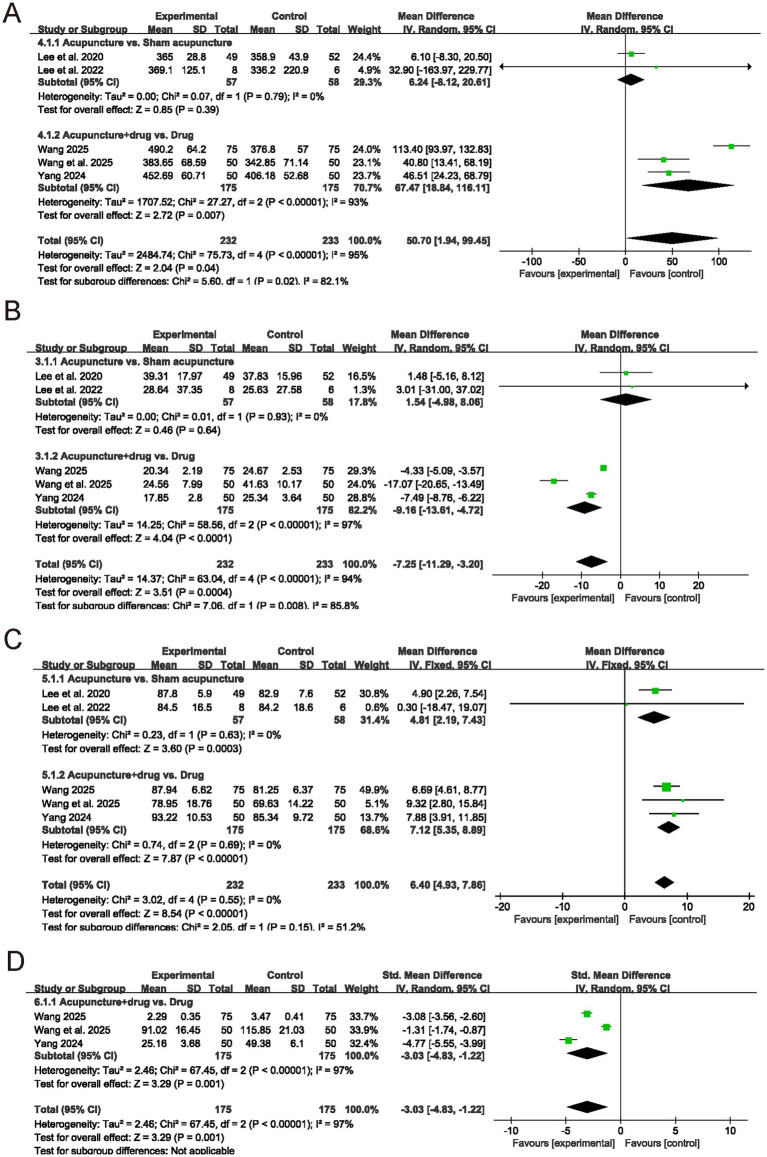
Forest plots for polysomnographic sleep outcomes. Forest plots of **(A)** total sleep time (TST), **(B)** sleep onset latency (SOL), **(C)** sleep efficiency (SE), and **(D)** wake after sleep onset (WASO).

For objective sleep parameters, several trials evaluated TST, SOL, SE, and WASO. As shown in Figure 6A, acupuncture was associated with a longer TST in the main analysis (MD = 50.70, 95% CI: 1.94 to 99.45, *p* = 0.04, *I^2^* = 95%), with the effect largely driven by acupuncture plus medication versus medication alone (MD = 67.47, 95% CI: 18.84 to 116.11, *p* = 0.007, *I^2^* = 93%), while acupuncture versus sham acupuncture showed no significant difference (MD = 6.24, 95% CI: −8.12 to 20.61, *p* = 0.39, *I^2^* = 0%). As shown in Figure 6B, SOL was reduced overall (MD = −7.25, 95% CI: −11.29 to −3.20, *p* = 0.0004, *I^2^* = 94%), again with a clear benefit in acupuncture plus medication trials (MD = −9.16, 95% CI: −13.61 to −4.72, *p* < 0.0001, *I^2^* = 97%) but not in sham controlled trials (MD = 1.54, 95% CI: −4.98 to 8.06, *p* = 0.64, *I^2^* = 0%). SE improved consistently (MD = 6.40, 95% CI: 4.93 to 7.86, *p* < 0.00001, *I^2^* = 0%) (Figure 6C), and WASO was reduced in acupuncture plus medication versus medication alone (SMD = −3.03, 95% CI: −4.83 to −1.22, *p* = 0.001, *I^2^* = 97%) (Figure 6D). Additionally, further subgroup analyses aligned with these findings (Supplementary Figures 4–7), showing improvements at 4 weeks for TST (MD = 29.66, 95% CI: 2.72 to 56.60, *p* = 0.03, *I^2^* = 73%) and SE (MD = 6.10, 95% CI: 4.03 to 8.18, *p* < 0.00001, *I^2^* = 0%), a borderline reduction in SOL (MD = −7.65, 95% CI: −15.36 to 0.06, *p* = 0.05, *I^2^* = 91%), and a reduction in WASO (SMD = −2.15, 95% CI: −4.12 to −0.18, *p* = 0.03, *I^2^* = 98%). Specifically, in chronic insomnia, effects were more consistent for TST (MD = 44.24, 95% CI: 26.95 to 61.52, *p* < 0.00001, *I^2^* = 0%) and SE (MD = 8.27, 95% CI: 4.88 to 11.66, *p* < 0.00001, *I^2^* = 0%), with reductions in SOL (MD = −12.13, 95% CI: −21.51 to −2.74, *p* = 0.01, *I^2^* = 96%) and WASO expressed in minutes (MD = −24.26, 95% CI: −26.17 to −22.35, *p* < 0.00001, *I^2^* = 0%), whereas analyses limited to two sessions per week did not show significant differences for TST or SOL. Collectively, these objective findings suggest that acupuncture may enhance sleep continuity and efficiency, with the largest and most consistent benefits observed in chronic insomnia and in trials using acupuncture as an adjunct to pharmacotherapy.

## 4 Discussion

This systematic review and meta analysis synthesized evidence from 12 randomized controlled trials and found that acupuncture based interventions were associated with improved sleep outcomes in patients with insomnia. Across studies, acupuncture reduced PSQI scores and improved polysomnography derived parameters, with the most consistent benefit observed for sleep efficiency, supporting a favorable effect on sleep continuity. Endocrine outcomes also pointed to potential modulation of circadian and stress related pathways, including higher serum melatonin overall, a more consistent melatonin signal in electroacupuncture trials, and lower salivary cortisol in the small number of studies reporting this measure. Although substantial heterogeneity was observed for several outcomes, subgroup patterns suggested that treatment intensity, add on designs, and insomnia subtype may contribute to between study variability. Taken together, these findings support acupuncture as a promising adjunct or alternative option for insomnia and provide mechanistic clues linking neuromodulation with HPA axis and circadian regulation, while underscoring the need for larger, standardized trials with harmonized endocrine sampling and objective sleep assessment to strengthen causal inference and clinical translation.

Insomnia is increasingly conceptualized as a disorder of hyperarousal involving dysregulation across neuroendocrine and autonomic systems rather than a purely behavioral sleep complaint (26). Clinical and experimental work links chronic insomnia to heightened activity of the hypothalamic pituitary adrenal axis and altered cortisol dynamics, supporting a stress system contribution to insomnia pathophysiology (27, 28). Within this framework, the endocrine related outcomes synthesized in the present analysis are biologically plausible indicators of upstream changes in stress and circadian regulation, but they require cautious interpretation because relatively few trials contributed to these analyses and measurement approaches were not fully standardized (28, 29). Melatonin is a key circadian signal that influences sleep propensity and circadian phase, and its measurement is highly sensitive to sampling time, light conditions, and assay characteristics, which can inflate between study variability when protocols are heterogeneous (29). In the present synthesis, serum melatonin increased after acupuncture based interventions in the overall pooled analysis, but heterogeneity was extreme, suggesting that protocol level factors and population differences materially influenced the observed endocrine signal (29, 30). A notable finding was that electroacupuncture showed a consistent and statistically significant increase in serum melatonin with no heterogeneity in the technique based subgroup analysis, which suggests that standardized electrical stimulation may yield a more reproducible endocrine response than manual techniques within the current evidence base (31). This observation is consistent with mechanistic work demonstrating that electroacupuncture can engage specific autonomic circuits, including pathways involving vagal related neuroanatomy, and that stimulation at defined body regions can differentially drive downstream physiological effects (31). Converging evidence also indicates that acupuncture can modulate hypothalamic pituitary adrenal axis related signaling, providing a mechanistic context for endocrine changes observed in clinical trials, even though direct endocrine endpoints were not uniformly measured across studies (32). Salivary endocrine outcomes were derived from a very small number of trials in the present dataset, and conclusions for salivary melatonin and salivary cortisol should therefore be viewed as exploratory and sensitive to sampling design and circadian timing.

Autonomic imbalance is another central feature of the hyperarousal model, and acupuncture has been reported to influence autonomic indices such as heart rate variability, although effects differ by point selection, stimulation parameters, and study context (33). The vagus nerve is a principal conduit for bidirectional gut brain communication and integrates visceral inputs into central autonomic and neuroendocrine networks, linking stress responses, inflammation, and behavioral states including sleep (34). The broader microbiota gut brain axis literature shows that gut microbes and their metabolites can influence neuroendocrine and neurotransmitter pathways, including tryptophan metabolism and serotonergic signaling, with downstream effects on stress responsivity and sleep relevant physiology (35). Within insomnia focused mechanistic syntheses, acupuncture has been proposed to act on the microbiota gut brain axis through combined modulation of autonomic tone, neuroendocrine outputs, neurotransmitter profiles, and inflammatory signaling, and electroacupuncture may exert stronger or more consistent neuromodulatory input because stimulation parameters can be standardized (36). This mechanistic interpretation is compatible with the direction of changes reported in several included trials, in which sleep improvement was accompanied by shifts in neuroendocrine and neurotransmitter related markers such as melatonin and cortisol, and in some studies GABA, serotonin, norepinephrine, or ACTH, although these biomarkers were not consistently assessed across trials and were measured using heterogeneous protocols (29, 32). However, the included randomized trials did not directly measure gut microbiota composition or microbiota derived metabolites, so microbiota mediated mechanisms remain inferential in this evidence base and represent a priority for future studies designed to test gut brain axis hypotheses.

Across the included trials, commonly used point sets clustered around GV20 with EX HN3 or GV29 together with HT7, SP6, and PC6, and these points have been repeatedly discussed in relation to central regulation, stress related symptoms, and sleep relevant neurobiological pathways (10, 37). Clinical commentary and evidence synthesis on HT7 in particular supports its relevance to insomnia and stress related symptoms, providing a rationale for its frequent inclusion in the protocols analyzed here (37). Experimental work using DU20, HT7, and SP6 based stimulation also supports effects on central signaling pathways linked to neuronal survival and plasticity in insomnia models, which is consistent with a multi pathway neuroregulatory mechanism (38). While these mechanistic links are plausible, the diversity of acupuncture prescriptions and dose components across trials means that cross study comparisons remain limited, and standardized reporting of dose parameters is critical for future mechanThe high heterogeneity observed across PSQI, melatonin, ISI, and several objective sleep measures likely reflects multiple interacting sources, including individualized acupuncture prescriptions, differences in control conditions, variation in treatment frequency and duration, heterogeneity of enrolled populations, and differences in outcome measurement methods and timing (30). In the present synthesis, subgroup patterns suggested that treatment intensity and clinical profile may modify effects, including frequency related differences in PSQI and more consistent objective improvements in chronic insomnia and in add on designs, which may partly explain between study variability (30). The credibility of pooled estimates is also shaped by study quality, and limitations such as incomplete blinding, reliance on self reported outcomes in some trials, and incomplete outcome data in a minority of studies can increase uncertainty and may inflate effects for subjective endpoints. At the same time, the observation that heterogeneity decreased and results remained directionally consistent in selected settings, such as electroacupuncture for serum melatonin and sleep efficiency outcomes with minimal inconsistency, supports higher confidence for these specific findings than for outcomes derived from few studies and heterogeneous protocols (29, 31). Accordingly, conclusions regarding endocrine outcomes should be stated cautiously and framed as signals that warrant confirmation in larger trials with harmonized sampling schemes rather than as definitive mechanistic proof. Formal publication bias assessment was not feasible for most outcomes and subgroup analyses because the number of contributing trials was generally below thresholds at which funnel plot based approaches and asymmetry tests are considered informative and adequately powered (39, 40). In addition, funnel plot asymmetry can arise from sources other than publication bias, including heterogeneity and small study effects, which further limits interpretability when study numbers are small and protocols are diverse (30, 39). Therefore, selective non-publication and small study effects cannot be excluded and should be regarded as additional sources of uncertainty when interpreting the pooled estimates (40). Future randomized trials should standardize acupuncture dosage and stimulation parameters, incorporate objective sleep measures alongside validated scales, harmonize endocrine sampling times across circadian windows, and include microbiota and metabolomic endpoints to directly test gut brain axis hypotheses and clarify modality specific effects.

## 5 Conclusion

Overall, this systematic review and meta-analysis suggested that acupuncture may improve insomnia related sleep outcomes and is associated with endocrine modulation, reflected by higher MT and lower CORT in the available evidence. Serum MT increased after acupuncture, and electroacupuncture showed the most consistent signal, whereas salivary MT did not differ and salivary CORT was lower in a small number of trials. These findings support acupuncture as a complementary option for insomnia related endocrine dysfunction and justify confirmation in larger, rigorously designed trials with standardized protocols and harmonized circadian sampling.

## Data Availability

The raw data supporting the conclusions of this article will be made available by the authors, without undue reservation.
