# Supplementary material for: Sleep and endocrine effects of acupuncture for insomnia: a systematic review and meta-analysis of randomized controlled trials
Source: Front Med (Lausanne). 2026 Apr 7;13:1807826. doi: 10.3389/fmed.2026.1807826 (PMC13095780; doi:10.3389/fmed.2026.1807826)
Supplement: Supplementary file 1 [file Data_Sheet_1.docx]

Supplementary Material

# Supplementary Figures


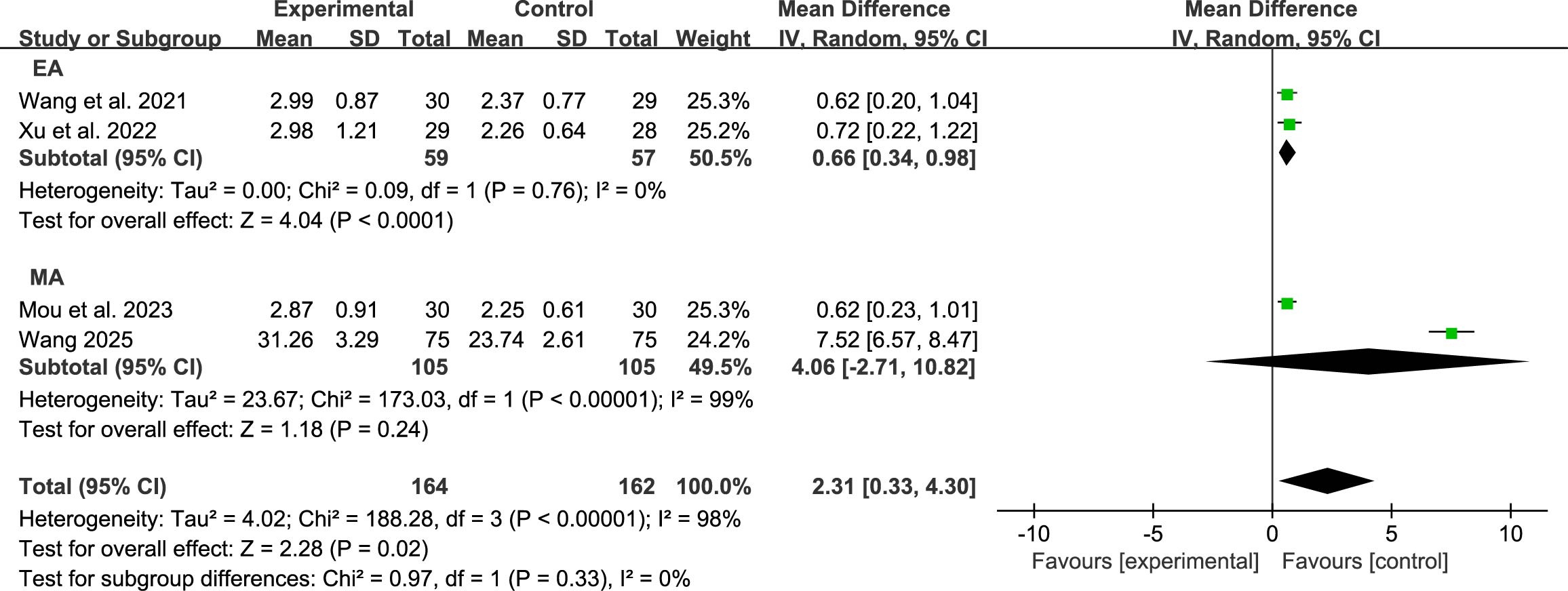


**Supplementary Figure 1.** Subgroup analysis of serum melatonin levels by acupuncture technique in randomized controlled trials. Forest plot comparing electroacupuncture (EA) and manual acupuncture (MA) versus control conditions, expressed as mean difference (MD) with 95% confidence intervals using a random effects model.


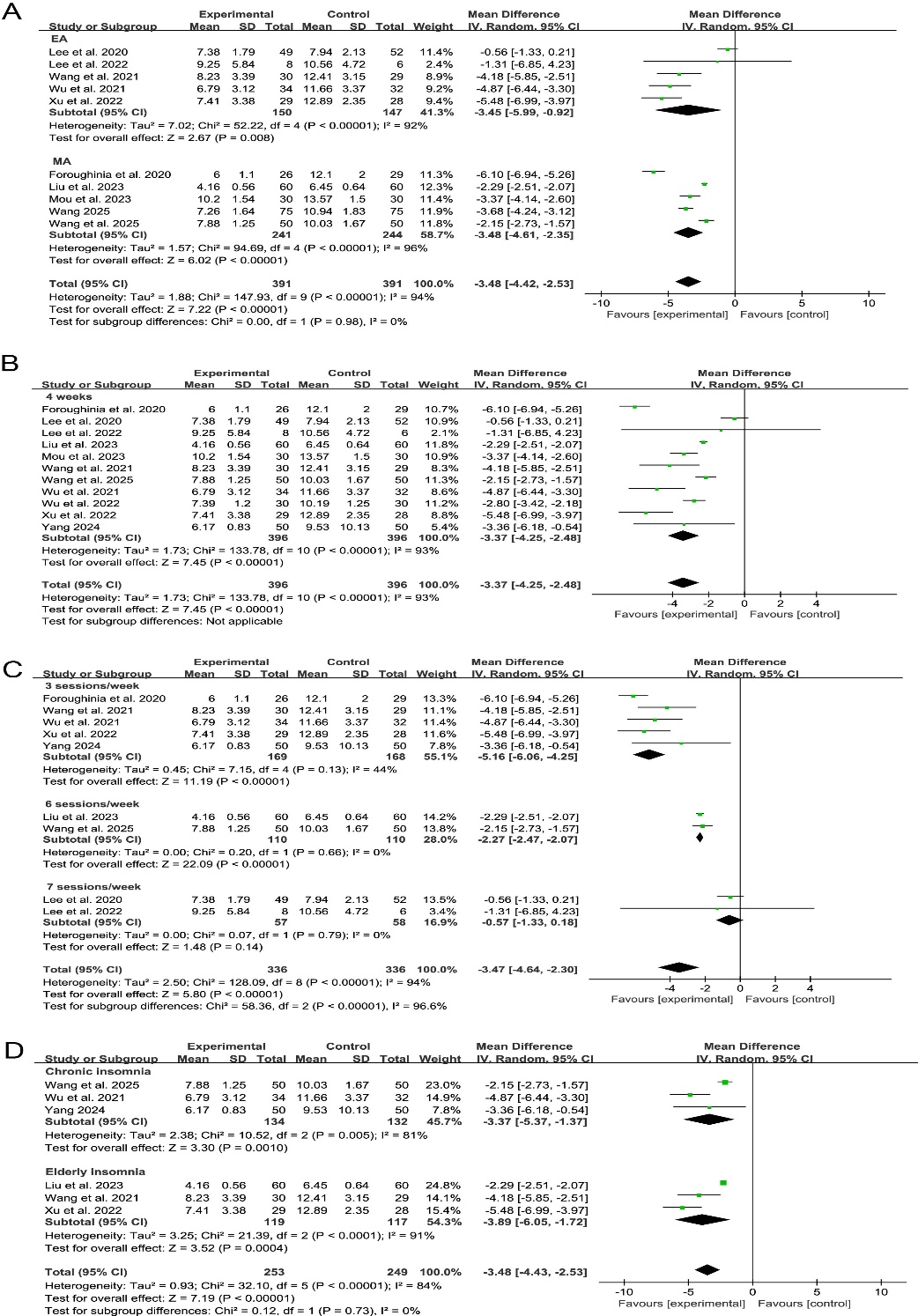


**Supplementary Figure 2.** Subgroup analyses of PSQI by intervention characteristics and insomnia subtype. Forest plots show the effects of acupuncture based interventions on Pittsburgh Sleep Quality Index scores, expressed as mean difference (MD) with 95% confidence intervals using a random effects model. (A) Subgroup by acupuncture technique, electroacupuncture (EA) versus manual acupuncture (MA). (B) Subgroup by treatment duration, 4 weeks. (C) Subgroup by treatment frequency, 3, 6, or 7 sessions per week. (D) Subgroup by insomnia subtype, chronic insomnia versus elderly insomnia.


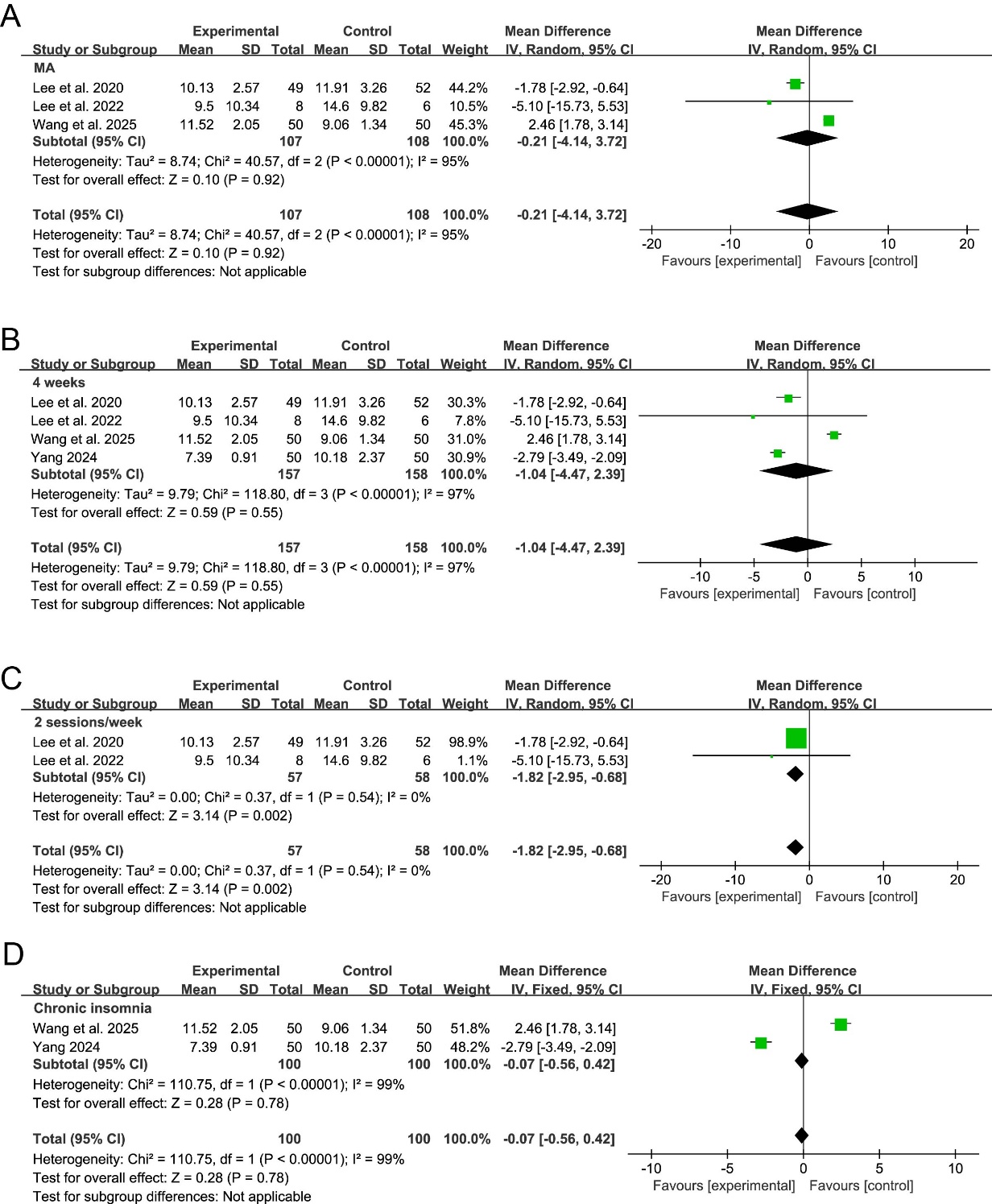


**Supplementary Figure 3.** Subgroup analyses of ISI by intervention characteristics and insomnia subtype. Forest plots show the effects of acupuncture based interventions on Insomnia Severity Index scores, expressed as mean difference (MD) with 95% confidence intervals. (A) Subgroup by acupuncture technique. (B) Subgroup by treatment duration, 4 weeks. (C) Subgroup by treatment frequency, 2 sessions per week. (D) Subgroup by insomnia subtype, chronic insomnia.


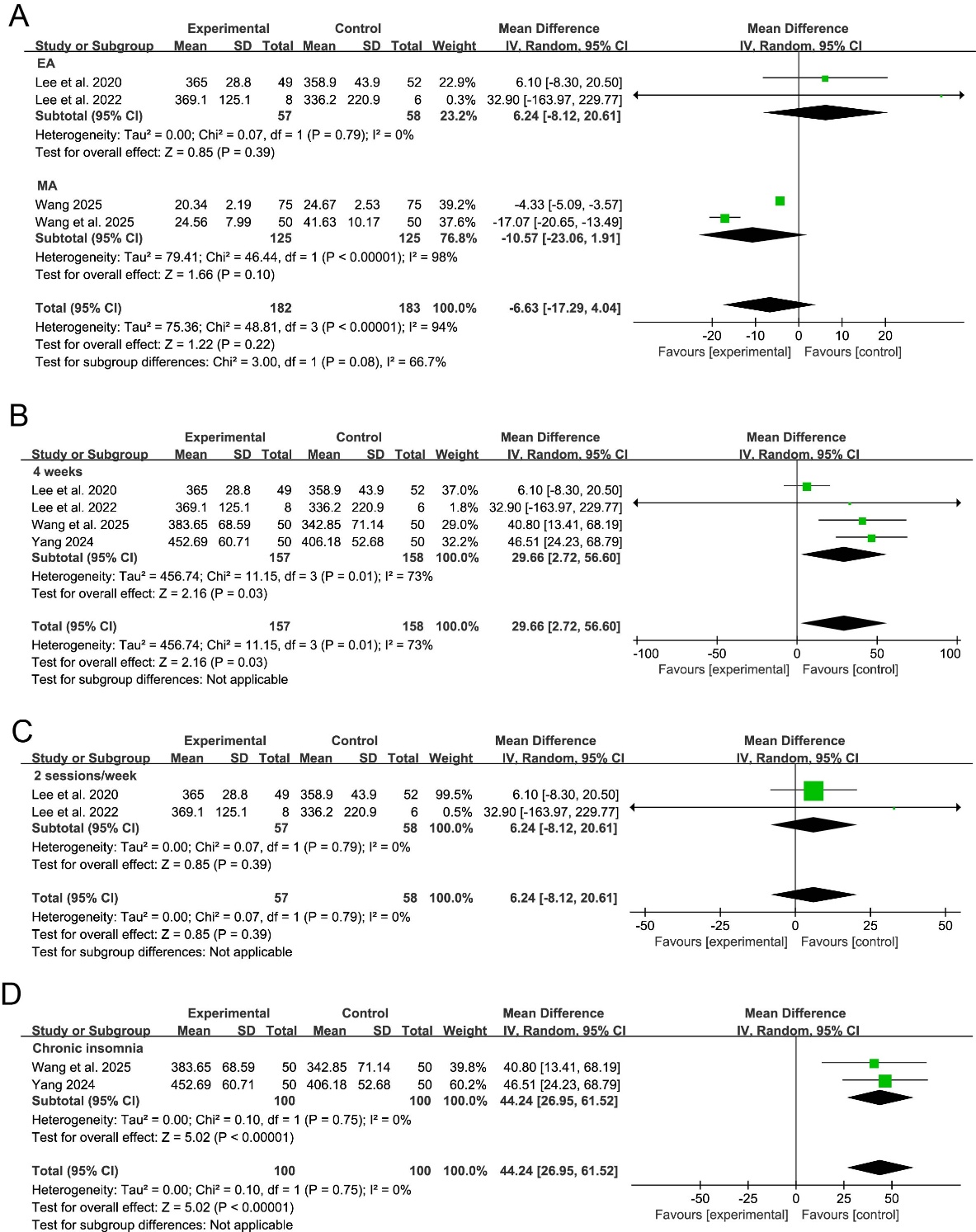


**Supplementary Figure 4.** Subgroup analyses of polysomnography derived total sleep time. Forest plots show the effects of acupuncture based interventions on total sleep time, expressed as mean difference (MD) with 95% confidence intervals. (A) Subgroup by acupuncture technique, electroacupuncture (EA) versus manual acupuncture (MA). (B) Subgroup by treatment duration, 4 weeks. (C) Subgroup by treatment frequency, 2 sessions per week. (D) Subgroup by insomnia subtype, chronic insomnia.


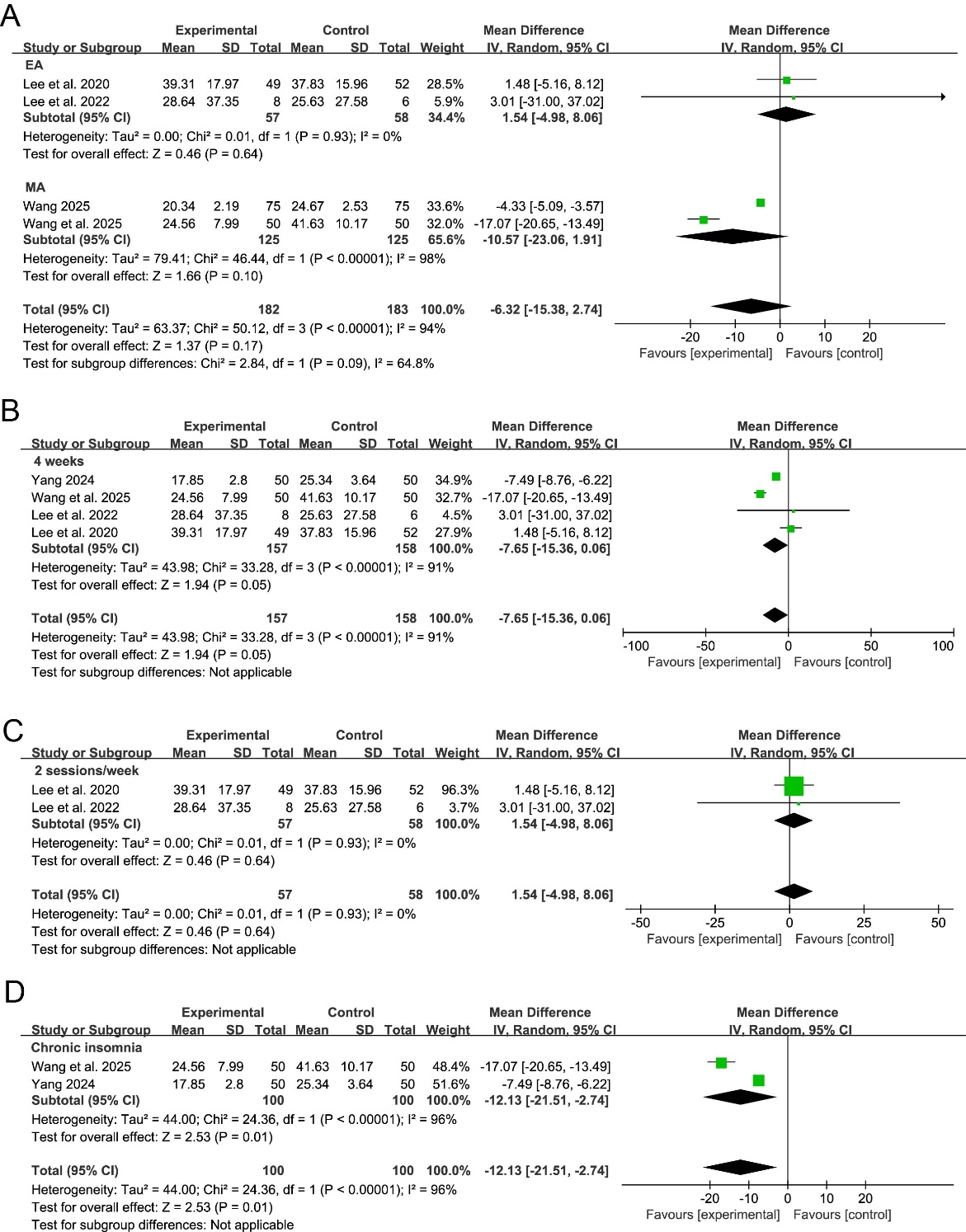


**Supplementary Figure 5.** Subgroup analyses of polysomnography derived sleep onset latency. Forest plots show the effects of acupuncture based interventions on sleep onset latency, expressed as mean difference (MD) with 95% confidence intervals using a random effects model. (A) Subgroup by acupuncture technique, electroacupuncture (EA) versus manual acupuncture (MA). (B) Subgroup by treatment duration, 4 weeks. (C) Subgroup by treatment frequency, 2 sessions per week. (D) Subgroup by insomnia subtype, chronic insomnia.


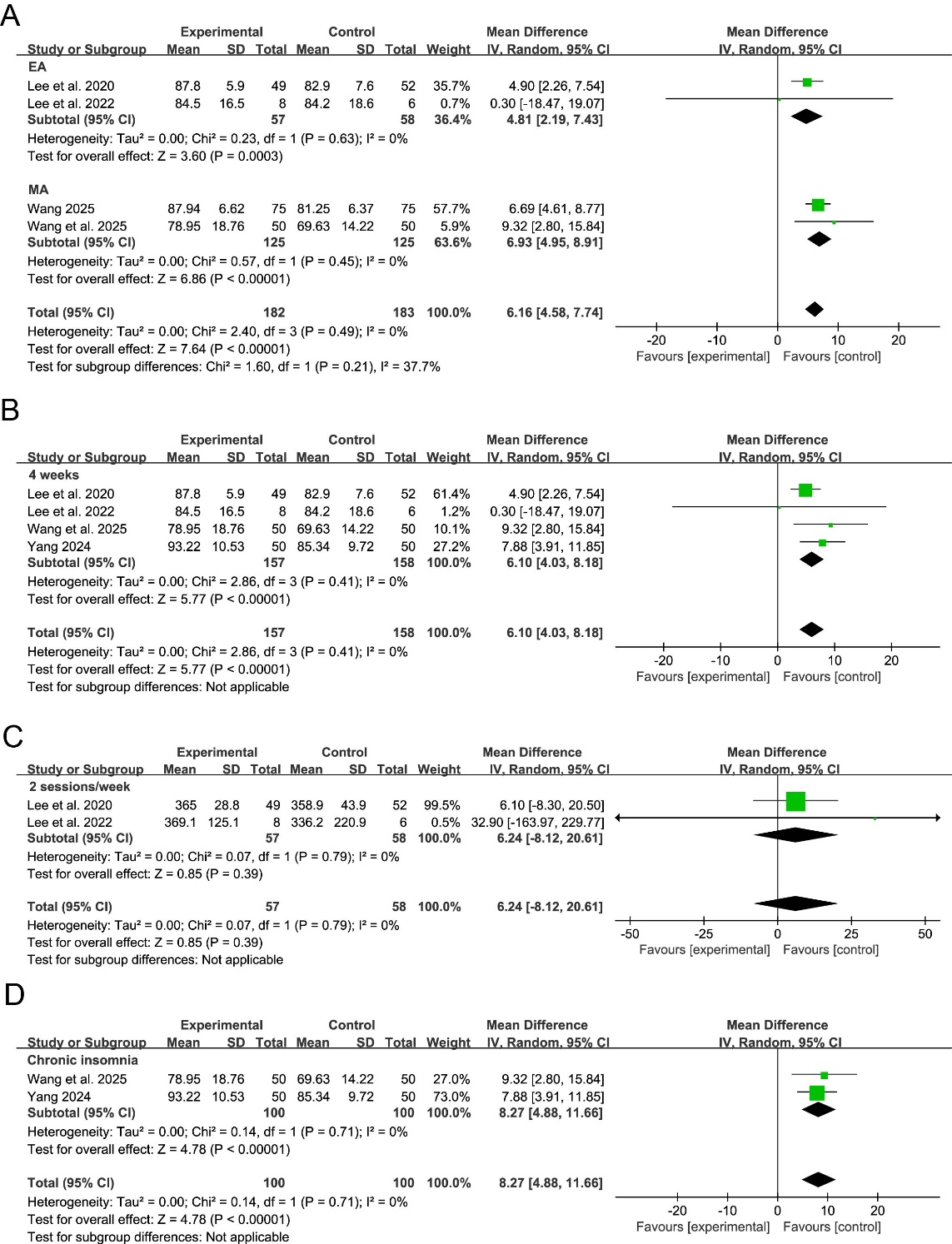


**Supplementary Figure 6.** Subgroup analyses of polysomnography derived sleep efficiency. Forest plots show the effects of acupuncture based interventions on sleep efficiency, expressed as mean difference (MD) with 95% confidence intervals using a random effects model. (A) Subgroup by acupuncture technique, electroacupuncture (EA) versus manual acupuncture (MA). (B) Subgroup by treatment duration, 4 weeks. (C) Subgroup by treatment frequency, 2 sessions per week. (D) Subgroup by insomnia subtype, chronic insomnia.


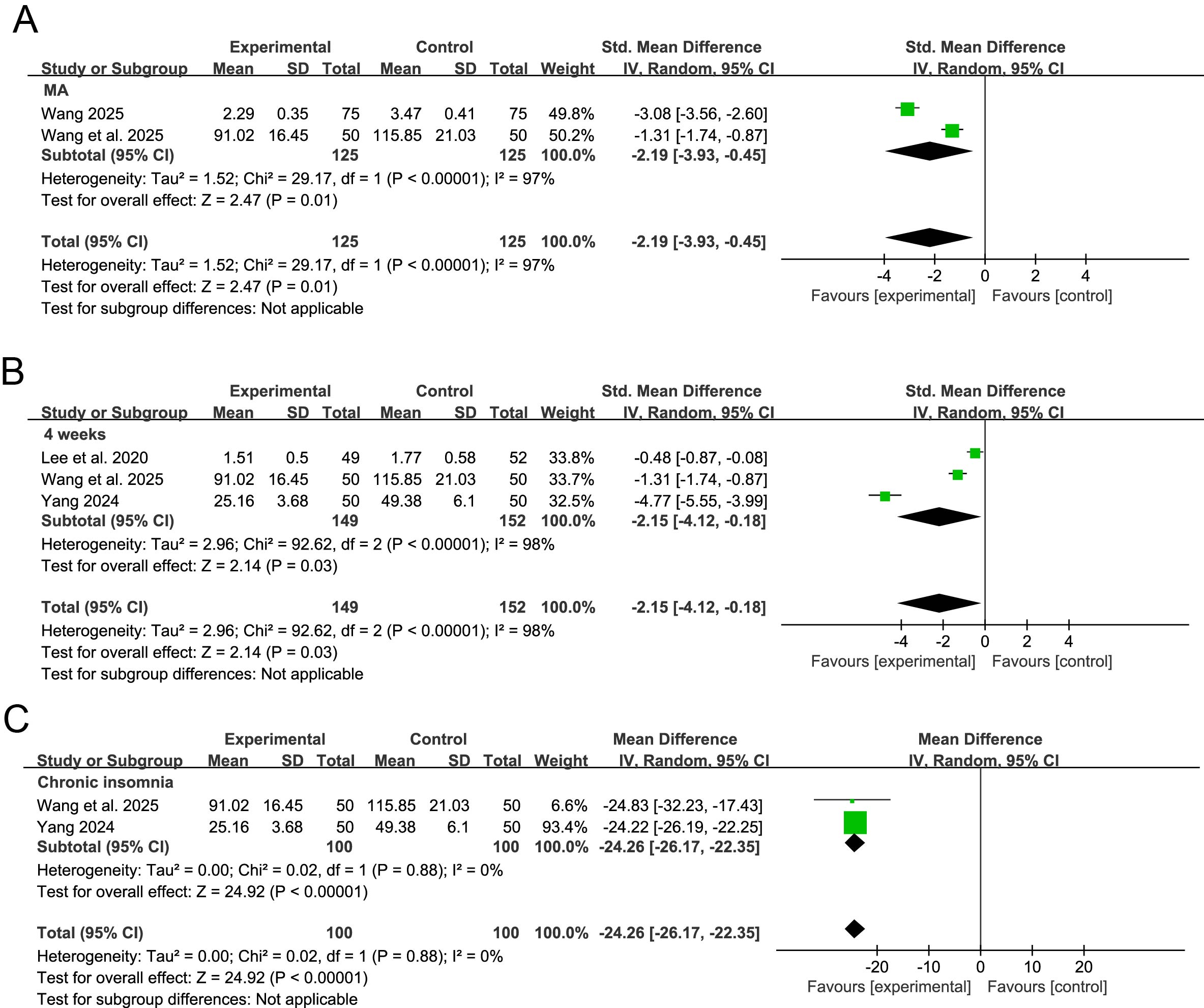


**Supplementary Figure 7.** Subgroup analyses of wake after sleep onset (WASO) measured by polysomnography. Forest plots display the standardized mean difference (SMD) with 95% confidence intervals using a random-effects model. (A) Subgroup by acupuncture technique (manual acupuncture, MA). (B) Subgroup by treatment duration (4 weeks). (C) Subgroup by insomnia subtype (chronic insomnia).

# Supplementary Tables

Supplementary Table 1. Search strategy.

| No. | Database | Search items |
| --- | --- | --- |
| 1 | PubMed | 1. ("Randomized Controlled Trial"[Publication Type] OR "Randomized Trial"[All Fields]) 2. ("Acupuncture"[MeSH Terms]) 3. ("acupuncture therapy" OR "acupuncture" OR "acupoint" OR "electroacupuncture" OR "electro-acupuncture" OR "warming needle" OR "auricular acupuncture") [All Fields] 4. or/2,3 5. ("Insomnia"[MeSH Terms] OR "sleep disorder" OR "sleep disturbance" OR "sleep quality") [All Fields] 6. ("Endocrine dysfunction" OR "hormones" OR "melatonin" OR "cortisol" OR "adrenocorticotropic hormone" OR "ACTH" OR "thyroid stimulating hormone" OR "TSH" OR "growth hormone" OR "GH" OR "prolactin" OR "sex hormones") [All Fields] 7. or/5,6 8. "humans"[MeSH Terms] 9. 1 and 4 and 7 and 8 |
| 2 | Cochrane Library | ("Acupuncture" OR "Electroacupuncture" OR "Acupuncture Therapy" OR "Acupoint" OR "Auricular Acupuncture" OR "Warming Needle")  AND ("Randomized Trial" OR "Randomized Controlled Trial")  AND ("Insomnia" OR "Sleep Disorder" OR "Sleep Disturbance" OR "Sleep Quality" OR "Chronic Insomnia" OR "Primary Insomnia")  AND ("Endocrine Dysfunction" OR "Endocrine Disorder" OR "Hormonal Imbalance" OR "Hormonal Regulation" OR "Melatonin" OR "Cortisol" OR "Adrenocorticotropic Hormone" OR "ACTH" OR "Thyroid Stimulating Hormone" OR "TSH" OR "Growth Hormone" OR "GH" OR "Prolactin" OR "Sex Hormones") |
| 3 | Embase | ("Acupuncture" OR "Electroacupuncture" OR "Acupuncture Therapy" OR "Acupoint" OR "Auricular Acupuncture" OR "Warming Needle")  AND ("Randomized Controlled Trial" OR "Randomized Trial" OR "RCT")  AND ("Insomnia" OR "Sleep Disorder" OR "Sleep Disturbance" OR "Chronic Insomnia" OR "Primary Insomnia")  AND ("Endocrine Dysfunction" OR "Endocrine Disorder" OR "Hormonal Imbalance" OR "Hormonal Regulation" OR "Melatonin" OR "Cortisol" OR "Adrenocorticotropic Hormone" OR "ACTH" OR "Thyroid Stimulating Hormone" OR "TSH" OR "Growth Hormone" OR "GH" OR "Prolactin" OR "Sex Hormones")  using a combination of multi-field search in all fields and Emtree |
| 4 | Web of Science | TS = ("Acupuncture" OR "Electroacupuncture" OR "Acupuncture Therapy" OR "Acupoint" OR "Auricular Acupuncture" OR "Warming Needle")  AND TS = ("Insomnia" OR "Sleep Disorder" OR "Sleep Disturbance" OR "Chronic Insomnia" OR "Primary Insomnia" OR "Sleep Quality")  AND TS = ("Endocrine Dysfunction" OR "Endocrine Disorder" OR "Hormonal Imbalance" OR "Hormonal Regulation" OR "Melatonin" OR "Cortisol" OR "Adrenocorticotropic Hormone" OR "ACTH" OR "Thyroid Stimulating Hormone" OR "TSH" OR "Growth Hormone" OR "GH" OR "Prolactin" OR "Sex Hormones")  Refined by: Document Types = ("Randomized Controlled Trial" OR "Clinical Trial" OR "Randomized Trial")  Timespan: All years  Indexes: SCI-EXPANDED, SSCI, A&HCI, CPCI-S, CPCI-SSH, ESCI |
| 5 | The Chinese National Knowledge Infrastructure Database (CNKI) | ("Acupuncture" OR "Electroacupuncture" OR "Acupuncture Therapy" OR "Acupoint" OR "Auricular Acupuncture" OR "Warming Needle")  AND ("Insomnia" OR "Sleep Disorder" OR "Sleep Disturbance" OR "Sleep Quality Decline" OR "Chronic Insomnia" OR "Primary Insomnia")  AND ("Endocrine Function" OR "Endocrine Dysfunction" OR "Hormone Level" OR "Melatonin" OR "Cortisol" OR "Adrenocorticotropic Hormone" OR "ACTH" OR "Thyroid Stimulating Hormone" OR "TSH" OR "Growth Hormone" OR "GH" OR "Prolactin" OR "Sex Hormones")  AND ("Randomized Controlled Trial" OR "Randomized Controlled Study" OR "RCT") |
| 6 | Wanfang | 1. ("Acupuncture" OR "Electroacupuncture" OR "Acupuncture Therapy" OR "Acupoint" OR "Auricular Acupuncture" OR "Warming Needle") [Common field] 2. ("Insomnia" OR "Sleep Disorder" OR "Chronic Insomnia" OR "Primary Insomnia") [Common field] 3. ("Endocrine Function" OR "Endocrine Dysfunction" OR "Hormone Level" OR "Melatonin" OR "Cortisol" OR "Adrenocorticotropic Hormone" OR "ACTH" OR "Thyroid Stimulating Hormone" OR "TSH" OR "Growth Hormone" OR "GH" OR "Prolactin" OR "Sex Hormones") [Common field] 4. ("Randomized Controlled Trial" OR "Randomized Controlled Study" OR "RCT") [Common field] 5. 1 AND 2 AND 3 AND 4 |

**Supplementary Table 2.** Study summary of key findings and methodological limitations of the included randomized controlled trials.

| Study | Findings | Limitations |
| --- | --- | --- |
| Wang (2021) | EA significantly reduced PSQI total score and component scores (SOL, SE, TST, and WASO) vs baseline;  Serum MT was higher than sham post-treatment | Single-center study with a modest sample size and short intervention period; no longer-term follow-up;  Endocrine markers were collected at a single morning timepoint only |
| Wang et al. (2025) | Acupuncture group showed greater improvements in PSQI and ISI;  Clinical response rate: 88.0% (acupuncture) vs 72.0% (drug-only);  Both groups improved SOL/WASO/micro-arousal index and increased TST/SE; acupuncture group outperformed drug-only on all listed PSG indices;  Greater reductions in TNF-α, IL-1β, CORT, ACTH and greater increase in GABA in the acupuncture group vs drug-only. | Blinding and allocation concealment procedures were not described;  Clinical efficacy was based on PSQI reduction rate (subjective composite), which may overestimate benefit;  Effect sizes/CIs and post-treatment follow-up were not reported, limiting clinical interpretability and durability assessment |
| Wu et. al (2021) | EA improved PSQI, FS-14, and lowered serum CORT; sham showed smaller improvements in selected indices  Improvements in EA group were maintained at 4-week follow-up (no clear decline vs post-treatment) | Short follow-up (4 weeks) and no objective sleep testing beyond questionnaire outcomes;  Generalizability to other insomnia subtypes remains uncertain |
| Foroughinia et. al (2020) | PSQI improved vs control at week 3;  Urinary 6-sulfatoxymelatonin increased vs control;  No serious acupuncture-related AEs; only needling pain reported | No long-term follow-up to assess durability;  Small analyzed sample at study end (n=55), limiting generalizability |
| Lee et. al (2020) | No Week-4 differences across outcomes except sleep-diary SE;  No serious adverse events | Minimal separation between EA and sham at the primary endpoint (Week 4), suggesting strong placebo/context effects;  Outcomes were mainly self-reported (plus sleep diary); no polysomnography/actigraphy;  Salivary hormones were measured only at baseline and Week 4, limiting interpretation of endocrine effects |
| Lee et. al (2022) | No significant within- or between-group changes were observed in salivary cortisol or salivary melatonin;  EA showed significant within-group improvements in ISI and PSQI at 4 weeks post-treatment;  Sleep diary parameters improved mainly in EA; actigraphy did not show clear between-group differences | Recruitment constraints (strict eligibility; non-cancer-specialized sites), limiting generalizability and scalability;  Low correlation between sleep diary and actigraphy-derived SE |
| Mou et. al (2023) | Higher clinical response rate in MA group vs control group;  MA group showed greater improvements in PSQI (total + multiple factors) vs control group;  MT increased in MA group and exceeded control group post-treatment;  Control group showed no meaningful MT change  No treatment-related adverse events reported | Expectancy effects may bias between-group differences, especially subjective outcomes;  Needs multicenter, larger-sample confirmation |
| Liu et. al (2023) | Higher overall clinical effective rate in MA+drug vs Drug group;  Greater improvement in PSQI over time (weeks 2 & 4) in MA+drug vs Drug group;  Better PSG changes in MA+drug vs Drug group (TST, REM, sleep latency, awakening time, sleep efficiency;  Larger pre–post changes in serum MT, NE, 5-HT, and also DA & GABA, in MA+drug vs Drug group | Restricted population (elderly; TCM subtype “liver stagnation transforming to fire”) may limit generalizability;  No post-treatment follow-up reported |
| Wu et. al (2022) | AP+drug group outperformed Drug group on PSQI;  Serum MT and leptin levels were significantly upregulated in the AP+drug group;  Total effective rate higher in AP+drug group | Small sample size;  No follow-up; durability of effects unclear;  Outcomes mainly subjective scales; lacked objective clinical endpoints/assessments |
| Wang (2025) | Higher overall clinical response in AP+drug vs Drug group after 8 weeks;  Greater improvements in PSQI/BRMS/SDS/SAS and PSG sleep parameters in AP+drug vs Drug group;  Serum 5-HT, MT, β-EP, and GABA increased in both groups, with larger increases in AP+drug | Blinding not described; outcome assessment may be prone to bias;  Adverse events/safety reporting not presented in the available report sections;  Durability of endocrine/sleep changes unclear |
| Wang (2021) | The experimental group showed greater improvements than control in PSQI/ISI/FS-14, PSG indices (TST, SOL, SE, WASO), and daytime brain function indices;  Post-treatment serum MT and 5-HT were higher, and dopamine was lower in the experimental group than in the control group;  The overall response rate was higher in the experimental group, and no treatment-related adverse events were reported | Short treatment/assessment window; no longer-term endocrine/sleep follow-up;  Blinding not described, placebo/performance effects cannot be excluded |
| Wang et al. (2025) | PSQI and FSS improved in both groups, with lower post-treatment scores in the EA group vs control;  Serum MT increased in the EA group and was higher than control after treatment | Only morning serum MT was measured; circadian MT dynamics were not assessed;  No long-term follow-up; durability of sleep and MT changes is unclear |
